# GRC-Sensing: An Architecture to Measure Acoustic Pollution Based on Crowdsensing

**DOI:** 10.3390/s18082596

**Published:** 2018-08-08

**Authors:** Willian Zamora, Elsa Vera, Carlos T. Calafate, Juan-Carlos Cano, Pietro Manzoni

**Affiliations:** 1 Department of Computer Engineering (DISCA), Universitat Politècnica de València, 46022 Valencia, Spain; elsa.vera@uleam.edu.ec (E.V.); calafate@disca.upv.es (C.T.C.); jucano@disca.upv.es (J.-C.C.); pmanzoni@disca.upv.es (P.M.); 2 Faculty of Computer Science (FACCI), Universidad Laica Eloy Alfaro de Manabí, 130802 Manta, Ecuador

**Keywords:** mobile crowdsensing, smartphone, machine learning, noise-sensing, smart cities, weka

## Abstract

Noise pollution is an emerging and challenging problem of all large metropolitan areas, affecting the health of citizens in multiple ways. Therefore, obtaining a detailed and real-time map of noise in cities becomes of the utmost importance for authorities to take preventive measures. Until now, these measurements were limited to occasional sampling made by specialized companies, that mainly focus on major roads. In this paper, we propose an alternative approach to this problem based on crowdsensing. Our proposed architecture empowers participating citizens by allowing them to seamlessly, and based on their context, sample the noise in their surrounding environment. This allows us to provide a global and detailed view of noise levels around the city, including places traditionally not monitored due to poor accessibility, even while using their vehicles. In the paper, we detail how the different relevant issues in our architecture, i.e., smartphone calibration, measurement adequacy, server design, and client–server interaction, were solved, and we have validated them in real scenarios to illustrate the potential of the solution achieved.

## 1. Introduction

The degradation of the environment is currently one of the most critical problems affecting human beings. Industrial development, the increase of vehicles in circulation, demographic expansion, and large urban concentrations, create a whole series of conditions that affect, to a lesser or greater extent, the quality of the environment [1]. One of these factors is environmental noise. Investigations carried out in this field [1,2] show that, at a certain level of noise, this can be a serious problem affecting health both physically and psychologically, with irreversible effects. Also, different organizations and countries have regulated and stressed the importance and direct relationship between the control of environmental noise and the life quality of the population [2,3,4]. In particular, they have set forth policies regarding the permissible noise levels for society in general.

Regarding this issue, there are traditional solutions [5] that measure acoustic pollution through the use of professional sound level meters [6] that, although of considerable cost and size, offer high precision and sensitivity. Typically, these measurement sessions are not flexible and cannot be configured, taking place only in a few available points, and for short time intervals. In addition to the current solutions for measuring noise pollution, the pervasiveness of smartphones, together with the increasing integration of new sensors (e.g., ambient light, accelerometer, proximity, etc.) has initiated a new paradigm called mobile crowdsensing [7,8].

**Figure 1:**
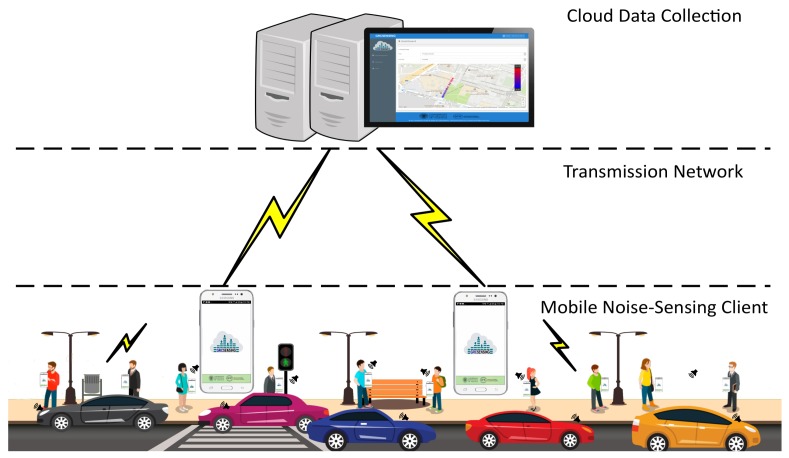
General architecture for noise assessment based on crowdsensing.

The concept of crowdsensing is that users of mobile devices participate by contributing some environmental data obtained through their devices, being these measurements then stored and subsequently handled using data fusion and data analysis techniques. The goal is to create a potential for data collection with great granularity in space and time. A general example of mobile crowdsensing for noise pollution analysis is shown in Figure 1.

In the literature, we find initial solutions that use smartphones to measure ambient noise in urban areas [9,10,11,12,13]. Many of these solutions have a participatory sensing [14,15] approach, detailing their architecture and integration with real-time collection solutions. Anyway, to the best of our knowledge, little research efforts have addressed the automatic reception of environmental noise collection tasks via smartphones.

Determining the sampling rate to be used, and the right time to gather the data by accounting for the context of smartphones, are both critical issues to consider when aiming at a widespread user adoption, as an excessive resource usage would make any application worthless. In fact, the sampling rate directly affects the use of the hardware resources in the smartphone, while smartphone context awareness is decisive to determine the best time and place to capture the noise, avoiding samples with limited or no representativeness. For example, it would be incorrect to measure noise when the smartphone user is playing music using the loudspeakers, or when the user is talking on the phone. Also, an extra factor to consider in the design of smartphone applications is battery power consumption. By avoiding to take noise samples at times where conditions are inadequate, it is possible to achieve significant energy savings, thereby extending the battery lifetime. Considering the issues mentioned above, solutions based on crowdsensing for ambient noise readings require new approaches to data collection that can reduce the level of authorization required by users and maximize energy efficiency. Another issue to consider is the impact of network usage in the process of recollection and distribution of sensing tasks within urban areas. Proposals such as [16] evaluate the performance of a vehicular crowdsensing application by assessing the number of packets delivered to road side units (RSUs) through realistic simulations in urban scenarios. In this paper, these types of issues are not tackled, and will be considered as future research.

We focus on application design issues; specifically, we attempt to detect which is the most appropriate time and context to obtain these noise samples, while simultaneously minimizing power consumption. Concerning the quality of the measurement, in a previous work [17] we describe the main characteristics that influence the design and implementation of candidate solution techniques for the assessment of noise pollution levels using smartphones. In this work, new processes related to (i) the precise time to capture the noise sample in an outdoor environment; and to (ii) achieving an optimization of the consumed battery resources, are evidenced.

The main contributions of our paper are: First, we propose an integrated architecture to measure noise pollution based on crowdsensing. In addition, we detail the main characteristics of each component our proposed architecture. Second, we propose a decision tree capable of simultaneously maximizing the precision of sampling decisions, while keeping time resource usage to a minimum. Third, we conduct the extensive experimental evaluation to demonstrate the significant performances of our decision tree. Finally, we validate our proposed architecture in real scenarios. Experimental results show that our optimized decision tree is able to reduce smartphone resource consumption by 60%. The actual scenario results show that our architectural proposal is adapted to a reliable and real-time solution for ambient noise sampling while introducing little development and deployment time overheads.

The rest of this paper is organized as follows: in Section 2 we present some related surveys on this topic. In Section 3 we provide an overview of the proposed architecture. Then, in Section 4, we define the contexts and algorithms used to determine the best sampling time automatically, and we describe the tool applied to obtain the candidate trees. Additional, we describe the procedure followed to take an alternative decision tree able to optimize resource consumption while maintaining the desired decision accuracy. Also, we describe the tests performed using a wide variety of smartphones models. The evaluation of our architecture in real scenarios is described in Section 5. Finally, in Section 6, we present our conclusions and future work.

## 2. Related Works

In the literature, we can find several solutions where smartphones are used as mobile sensing devices to make noise pollution measurements. For instance, works such as [9,10] propose noise-sensing solutions where the developed applications include a real-time (only smartphone) sound-level data logger that also includes Global Positioning Systems (GPS) data to generate a map. However, these works fail to provide details about the sensing task itself.

NoizCrowd [12], and Ear-Phone [11,18] study noise levels using different spatial and temporal interpolation techniques. In particular, Ear-Phone [18] proposes an algorithm that attempts to optimize noise sampling by detecting when the phone is placed in the trouser’s pocket, or in a bag. NoizCrowd [12] uses GPS to determine the users’ locations, but fails to determine when and how the collection task is performed.

SoundOfTheCity [13] is a proposal that uses several sensors to provide context-awareness. This context-awareness allows distinguishing between situations where the user is located, outdoor, indoors, moving, or if the smartphone is in the user’s pocket (using GPS, Wi-Fi, and proximity) in order to determine the right instant to trigger the measurement. Later, NoiseSense [19] proposed a semi-supervised sensor completion algorithm for inferring noise levels for locations in an urban area where smartphone users are unable to provide measurements. This article does not present details on the data collection process, focusing solely on the sending of raw data to a server.

In general, previous solutions provided a few of the processes associated to data collection for environmental noise-sensing at a specific time and place. Alternatively, Usense [20] presented a generic middleware for developing and deploying crowdsensing applications. Also, it added a module that evaluates the precise moment of capture using a rule-based approach. This solution does not present specific details regarding the noise calibration process, nor does it describe or evaluate the windows size used for data collection.

In [21] authors describe a platform based on MCS called City Soundscape, which allows the use of smartphones (Android) as a tool for portable acoustic monitoring. Mobile users collect acoustic measurements through built-in microphones on smartphones and then, through a web-based application, provide information to city managers to enable corrective measures to be taken whenever appropriate.

Our work, in addition to adding new features, differs from the previous ones by focusing on optimizing the sensing procedure to minimize resource consumption. Besides, we have proposed a unique crowdsensing architecture for reading ambient noise, which differs from previous proposals by requiring minimal user intervention and allows the display of real-time heat maps. Also, our solution offers an automatic module to distribute tasks to mobile devices, allowing to capture environmental noise only in specific urban areas, and at specific times/dates. In Table 1 we show characteristics of existing solutions in the literature regarding the elements related to smartphone, transmission, and task specification.

Since energy efficiency is a critical issue for smartphones, several authors have addressed energy-efficient solutions. In [22] authors use a predictive model to find the optimal time interval to perform the sensing task. This module, called sensing decision engine, contributes to the overall cost-reduction. In [23] a middleware for crowdsensing is proposed where the main idea is that it suppresses the transmission of sensor readings from smartphones, and filters out the data which is not needed by the application logic. Other works [24,25,26] propose to reduce energy consumption by focusing on the use of GPS. In [27] the authors described a power modelling and measurement methodology to achieve model-based energy profiling for smartphones.

Regarding classification algorithms, in [28] the authors introduce a crowdsensing framework for location recognition. Specifically, they use algorithms based on Hidden Markov Models, as well as Gaussian models, for speech recognition and sound classification, respectively. Works such as [29] show how classification algorithms can be used for human activity recognition systems using wearable sensors. Shoaib et al. [30] present a review of the works using recognition systems on smartphones based on their on-board sensors. The works surveyed presented some considerations when designing applications having sensor-based activity recognition. In particular, we observed that, from the 30 reviewed papers, 60% of them did not address battery consumption analysis, and only 27% of them have performed a Central Processor Unit (CPU) usage analysis.

In view of the previous research studies, it becomes clear that, in spite of the many advances in the field of mobile crowdsensing in recent years, there are still a number of issues that must be addressed adequately for solutions to become more effective and, from the user perspective, it is clear that these tasks should not become a burden. Therefore, it is necessary to consider new crowdsensing architectures; in particular, proposals that include the management of context assessment to accelerate the discarding of unwanted samples. The energy consumption and network resources are also critical issues that can be carefully managed by intelligent algorithms capable of correctly determining the best sampling times, while simultaneously avoiding CPU-intensive tasks.

## 3. Crowdsensing Architecture Overview

**Figure 2:**
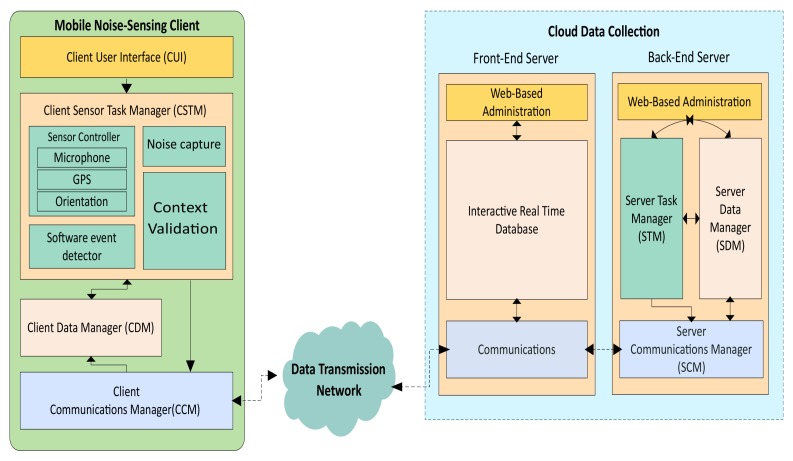
Proposed crowdsensing architecture for mobile noise analysis.

In a previous work [36] we have presented a generic client–server architecture for crowdsensing solutions. The main difference towards our present work is that we actually propose a global crowdsensing solution that focuses exclusively on noise-sensing in urban environments, where the different components are described, from the web platform to the capture process details. In particular, our proposed architecture defines a set of elements that can be adapted to any crowdsensing task, which in the scope of this work is monitoring the environmental noise in real time. Our design includes both the Mobile Noise-Sensing Client (MNSC), and the Cloud Data Collection (CDC). Those two elements are connected to each other through a data transmission network. The data transmission network works on the full scope of our architecture, and it provides support for iterative CDC and MSNC. In general, the MNSC is composed by mobile phones that will provide sound sensing operations by capturing noise data which will be delivered in real time to the CDC. In addition, the CDC can be a single server or a server farm that allows receiving, processing, analyzing and sharing the sensed data. Our complete architecture is shown in Figure 2. In the following part, we will detail the proposed architecture.

### 3.1. Mobile Noise-Sensing Client (MNSC)

In this section, we detail the client-side solution. Our solution allows the ambient noise to be sampled with little user intervention. Once activated, the mobile application seamlessly integrates and autonomously performs the noise reading through a service running in the background. Besides, once the smartphone is in the desired context, the noise is known and forwarded to the server for further processing. Figure 2 shows the main components of the Mobile Noise-Sensing Client. These are Client User Interface (CUI), Client Sensor Task Manager (CSTM), Client Data Manager (CDM), and Client Communications Manager (CCM). Each of these four components has a controller responsible for supporting either bidirectional or unidirectional interactions between the different system elements. We now proceed to describe each of the client components in detail.

#### 3.1.1. Client User Interface (CUI)

This component allows applications to interact with the user, and allows us to configure the different permissions associated with the operating system, such as: storage, audio, and location. After a startup, the splash screen closes and the main service is activated. Once the service has been activated, and the necessary permissions have been enabled, the application switches to the background, and the graphical interface is cleared from memory.

#### 3.1.2. Client Sensor Manager Task (CSTM)

The CSTM is the main component responsible for the noise-sensing task. In general, the process is the following: once the CSTM is activated, and the server provides a new task, the CSTM is responsible for validating the different environments where the smartphone is located. Once this is done, the noise value is read and then sent to the server. The CSTM has three sub-components: Context Validate, Sensor Controller, Noise capture, and Software Event Detector. Below we describe each of its sub-components.

- Context Validate—This sub-component aims to determine the optimal strategy for collecting noise pollution data through smartphones. In general, it integrates an algorithm-based decision tree that interacts synchronously with the Sensor Controller (SC) and the Software Event Detector (SED). The purpose of the interaction with CDM is to read the data of the task that is stored in the local database (SQLite). Regarding the interaction with SC and SED, its function is to obtain the previously processed values of the sensors (i.e., GPS, Orientation, etc.), and to perform calls to the operating system (i.e., determine whether the phone has the music player active), respectively. Once the answer is true for all cases, the algorithm proceeds to sample the noise. Also, the Context Validate has an algorithm that balances the use of tasks that are in the same range of date and time. Besides, to minimize energy consumption, Context Validate enforces a minimum time between trial and collected samples. Those times are given in the task from the CDC. In Section 4, we show the implementation details of our collection strategy.
- Sensor Controller—This component implements the access to the different sensors proposed in our architecture, thus providing access to gyroscope, accelerometer, microphone, and GPS.
- Noise capture—This component allows the data to be preprocessed before interacting with Validate Context. In particular, we process raw sound data and then store only a numeric dB(A) value to protect the privacy of users. In Section 4 we show the details and strategies to obtain a correct and calibrated measurement.
- Sensor Event Detector—This component allows to determine the different status of the smartphones (i.e., playing music, speaker on, and smartphone performing active call) through a call to the operating system.

#### 3.1.3. Client Data Manager (CDM)

The CDM performs two functions: (i) it allows us to store the tasks provided by the server; and (ii) it supports Structured Query Language (SQL) queries to the CSTM. In particular, the tasks are received in one direction by the CCM. Also, to avoid data redundancy, the tasks are previously consulted and, if they do not exist, then they should be stored. Once the tasks are registered, they are available through a query based on the starting date and time, and the final date and time for the CSTM.

#### 3.1.4. Client Communications Manager (CCM)

The main purposes of this component are interaction with the server such as receiving the tasks and forwarding the captured noise data to the server (CDC). In the first task, once the listener service is activated, the tasks on the Front-End Server (Interactive real-time database) are replicated for each mobile device that is registered in the Front-End Server. Each new task is verified for later storage in the local database. In the second case, the Front-End Server automatically offers the synchronization option between MNSC and Back-End Server. Figure 2 shows the direction of the communications that the proposed architecture components use.

### 3.2. Cloud Data Collection (CDC)

The CDC is our server-side solution. The server provides a web interface that allows the administrator to control the tasks, and it also allows us to have complete access to the information regarding trace management, processing and visualization. Once logged in, the administrator can create a new collection task, or display the noise data provided by smartphone clients using heat maps. The architecture is composed of two servers: (i) Front-End Server; and (ii) Back-End Server, as shown in Figure 2. In general, the Front-End Server acts as an intermediary between the Back-End Server and the MNSC, while the Back-End Server provides the processing and delivery of collection tasks. Below we provide more details about the specified components.

#### 3.2.1. Front-End Server

The Front-End Server is responsible for carrying out the communications for sending/receiving data from mobile devices and the Back-End Server. In general, it performs the intermediary functions relating the proposed components. In particular, once the Back-End Server provides the task, it is replicated to all client devices that have the application activated. In contrast, once the clients supply the noise data, this is automatically sent to the Back-End Server. In our architecture, the Front-End Server includes three components: (i) Web-Based Administration; (ii) Interactive Real-Time Database; and (iii) Communication; these components are described below.

**Figure 3:**
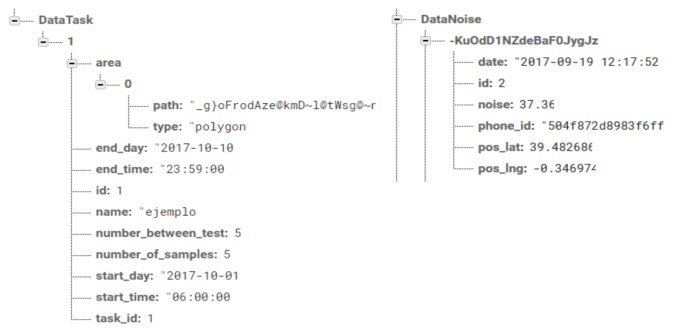
Format of a JSON data message.

- Web-Based Administration—It is the administration console provided by Firebase [37]. Firebase is a Google solution that is integrated with our architecture in a simple and transparent manner through their Application Programming Interface (API). Among other options (i.e., hosting, analytic, etc.), it allows us to manage the database in real time.
- Interactive Real Time Database—It is our real-time (NoSQL) database, whose format is JavaScript Object Notation (JSON). In general, it is a gateway responsible for automatically sending/receiving sensing tasks towards smartphones. The tasks are previously defined in the Back-End Server, and are sent to this database whenever the administrator user requires it. The data sent and received are temporarily saved for the duration of the date range defined in each task. In particular, we maintain two “*DataNoise*” and “*DataTask*” objects within our JSON object. The first one stores the values of the task, and the second one stores the values of the captured noise. Figure 3 shows the attributes of our JSON objects.
- Communications—Firebase uses a push communications model for sending data to specific recipients registered in its database. Generally, Firebase maintains a two-way open socket-based communications channel for the CDC and MNSC.

#### 3.2.2. Back-End Server

The Back-End Server provides a web interface which allows the administrator to have full access to the information gathered concerning trace handling, processing, and visualization. Additionally, it allows us to define, schedule, and store the noise-sensing data collection task. The Back-End Server has four components: Web-Based Administration, Server Task Manager (STM), Server Data Manager (SDM), and Server Communications Manager (SCM). Below, we describe in more detail the different components on the server side.

**Figure 4:**
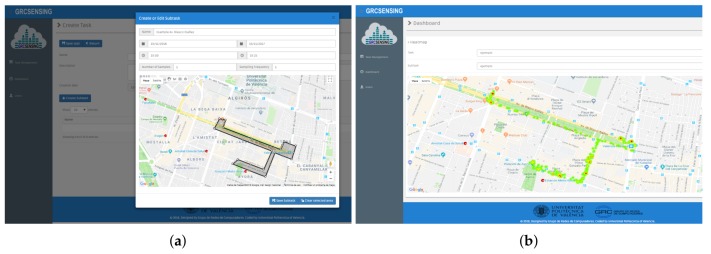
GRCSensing Web-based application. (**a**) Area definition using a polygon of n sides.; (**b**) Example of a heatmap detailing the noise distribution.

- Web-Based Administration: This component allows the user to manage and schedule noise-sensing tasks interactively. It also supports the visualization of charts (heat-map) relative to the sensed data. Both functionalities are performed using a web-based graphical interface, meaning that the system manager can operate remotely. The site is available at http://www.grcsensing.net, and its design is shown in Figure 4, where the administrator (among other users) can create sensing tasks in specific areas, as shown in Figure 4a,b; in the latter, an example of a heatmap in the previously defined area is shown.
- Server Task Manager (STM): Task Management is one of the main components of the Back-End Server according to our proposed architecture, being responsible for scheduling planning, and the pushing of crowdsensing noise tasks. For the definition of the tasks, we have created two attributes: one for the waiting time between attempts, and another one for the time between samples. The purpose of these features is to minimize the consumption of resources in the tasks handled by smartphones. Also, we have enabled three types of geographic areas selection (polygons of n sides, rectangle, and circle) for the capture of environmental noise. Finally, once created, the tasks are stored by the SDM, and they can be forwarded to the Front-End Server when the user administrator considers it necessary. Figure 4 shows an example to create the task for the gathering of noise, and the area selection using a circle.
- Server Data Manager (SDM): This component is responsible for the processing, storage, query, and analysis of the noise-sensing task.
- Server Communications Manager (SCM)—The SCM is the Rest API that supports the communication between the Back-End Server and the Front-End Server. In our architecture, we used a unidirectional interaction between SCM and STM, and a bidirectional one between SCM and SDM. The interaction with STM is unidirectional since we have transmission towards the Front-End Server when pushing new noise-sensing tasks. The communication with the SDM is bidirectional since, when the API is notified of the existence of a new registry (data capture), it is first consulted before being inserted. Once the record has been inserted into the database, the SCM proceeds to delete the record in the Front-End Server. In particular, we have used Pyrebase [38], which is an API written in Python. Figure 2 shows the communication between these components.

### 3.3. Data Transmission Network

This is the element responsible for the actual communication between the Cloud Data Collection (CDC) and the Mobile Noise-Sensing Client (MNSC) devices through the establishment of end-to-end connections. Typically, reliable TCP connections are established. In particular, we use the Firebase API, which supports high-level communications, by automatically opening sockets. At the client side, Firebase establishes its communication through generic sockets, so that it guarantees compatibility with all smartphones, while at the server the connection relies on a REST service, which specifically uses the “Pyrebase” library, a simple Python wrapper for the Firebase API. Additionally, Firebase includes in its API the persistence option on disk, which means that, if the mobile device loses the network connection, Firebase will cache the captured noise registers and, when the connection is again available, it will synchronize the data that was previously cached with the server. Finally, Table 2 shows the size of the message when the clients send data (DataNoise), and when the server sends the task (DataTask). We have used a Google library to encode and decode polygons in an n-sided data stream. This means that the adoption of this type of selection has little impact on the overall transmission load.

### 3.4. Implementation

Our MSNC app, called GRCSensing, was developed using Android Studio 6.01. Besides, a set of Google dependencies (i.e., maps, firebase) have been used for the coding and decoding details of GPS positions and Firebase APIs. Once the mobile application is installed, and the required permissions are granted, it automatically starts capturing ambient noise data. Those samples are sent in real time to the CDC. We use SQLite as the local data structure for storage.

Regarding the Server solution, specifically the Back-End Server, it is designed following the Model View Controller paradigm, and using the Django platform [39]. Also, we make use of JavaScript to add several functionalities, like those using Google maps, and to draw the different types of areas required. The database used is MariaDB, and the Pyrebase API is used for communications. For statistical analysis and reporting, we use the R Graph tool [40], which includes the generation of heat maps for captured noise.

## 4. Sampling Process Optimizations

During the design process of the mobile app, the developer defines different procedures to determine, e.g., when the sensors should be activated. Besides, certain conditions should be considered when using smartphones as a noise measurement tool. For instance, several situations make it inadequate to sense environmental noise, as user intervention is affecting the overall result. Examples of such situations include: talking on the phone, listening to music using the loudspeaker, or keeping the smartphone in a pocket/purse. On the other hand, a correct and calibrated reading of the values obtained by the smartphones is indispensable. In this section, we present the process we followed to ensure a suitable context for noise capture, and detail the calibration procedure used to make sure that the samples obtained are accurate. Both these elements are implemented as part of the Client Sensor Manager Task (CSMT).

### 4.1. Attribute Context Classification

In this subsection, we seek to assess the adequacy of a particular situation to take environmental noise samples. Specifically, we want to optimize the sequence followed to access the different built-in states and sensors (i.e., GPS, accelerometer) for such assessment to be made. We define a series of attributes that contribute to determining whether the ideal conditions for environmental noise sampling are met. These attributes are shown in Table 3, and were classified according to the conditions and characteristics of the smartphone in three different categories. For the first category, we consider the tasks as a set of actions to be taken by the smartphone at a specific time and location with the aim of measuring noise pollution (e.g., measure the noise level in the downtown area of Valencia, on Sunday the 11 October, between 12:00 and 13:00). In particular, it defines the task of sensing. Regarding the second category, it refers to the attributes that produce a sound phenomenon, and that can cause the noise reading to be inaccurate. The third category, refers to the conditions of the smartphones (e.g., the smartphone can be active or idle). These two last categories refer to the optimal instant to perform the noise measurement. The attributed associated to the different task categories are detailed below:

- TaskDate. This attribute allows you to validate the existence of a new sensing task pushed by the server, and to check the range of dates and times associated with a particular task.
- TaskArea. This attribute allows determining whether the smartphone is within any of the target areas considered of interest to the task. In this study, we have defined the target area as a generic polygon of n sides, or as a circle.

Proposed attributed for the sound category:

- Speaker. This attribute allows checking whether the smartphone’s built-in speakers are on or off. We make use of a call to the system audio administrator to obtain this state information.
- Music. This attribute allows determining when the smartphone is playing music. This information is made available through a call to the operating system. Such playback can be triggered by an event produced by WhatsApp, Spotify, Youtube, or similar applications.
- ActiveCall. This attribute allows determining whether the smartphone has an active call through the telephony management API, which allows determining the specific state of the device.
- PhoneStatus. This attribute refers to the four main states of a smartphone: on the hand, on a flat surface facing upwards/downwards, in a pocket, and in a bag. If the smartphone is being held, or if the smartphone is on a flat surface facing upwards, this is considered a favorable context. We have discarded the options where the smartphone is inside a bag, in a pocket, and over a flat surface facing downwards. Section 4.2 provides details about the classification method adopted for PhoneStatus.
- ActiveApplication. This context allows determining whether the smartphone is making use of some type of social network application or similar (i.e., WhatsApp, Instagram, Facebook, etc.).

Proposed attributed in the status category:

- Block. This attribute allows determining whether the smartphone’s screen is locked or unlocked.
- Microphone. This attribute allows determining whether the microphone integrated in the smartphone is activated or not.
- KeyBoard. This attribute allows determining whether the screen keyboard is activated or not. In general, this can be an indicator that the user is actively using an application, and it can be a good moment to perform a noise sample.
- Camera. This attribute allows determining whether the smartphone’s camera is activated or not.
- Location. This attribute allows determining whether the smartphone is indoors or outdoors, as only outdoor measurements are targeted. This is assessed by accounting for the satellite visibility, which is quite reduced when indoors.

Overall, a smart combination of the aforementioned attributes should allow an adequate assessment of the adequacy of sampling conditions. Also, notice that the first two attributes (TaskDate, and TaskArea) are in fact defined by system managers themselves using the cloud server application. So, each time a user is notified about a new crowdsensing task, the application should collect these attributes, validating and processing them. Based on these 12 nominal attributes defined above, we have created a list of all possible combinations (16,384 cases), being all of them tagged as admissible or not from the perspective of environmental noise assessment.

**Figure 5:**
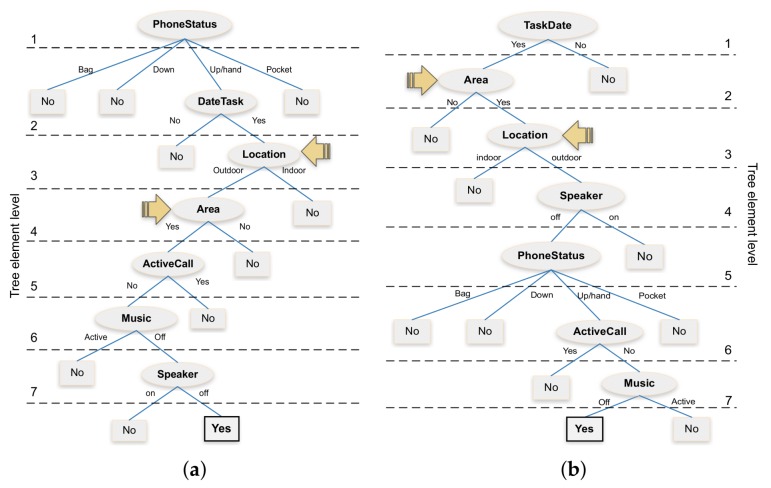
Visualization of the automatically generated trees. (**a**) J48 algorithm; (**b**) Random Tree algorithm.

Using as input the 16,384 cases referred above, we relied on the Weka tool [41] to provide an automatic classification of these different cases. As an output, Weka generated two decision trees, one using the J48 algorithm [42], and another one using the RandomTree algorithm [43]. Figure 5 shows the obtained decision trees. Regarding their accuracy, the J48 algorithm achieves a 100% accuracy, while for the RandomTree algorithm, the accuracy achieved is slightly reduced (99.89%), with an absolute error of 0.001. Overall, it is worth mentioning that attributes *ActiveApplication, Block, Microphone, Keyboard*, and *Camera* have been discarded, as they are considered unnecessary by both algorithms.

From a resource consumption perspective, we can observe that the location-related attributes are positioned in the fourth node of both trees, as signalled by the beige arrows. This leads us to think that the resource consumption associated with these decision trees can be excessively high. Additionally, for our study, the “*TaskDate*” attribute should be considered at the beginning of each tree since this is a basic requirement to check for the existence of a new task. In short, these two proposed decision trees offer a viable theoretical solution, but they are not optimal from a software design perspective, and they are not at all optimal regarding time and energy consumption. So, in the next section, we will analyze the different issues involved to optimize the decision process properly; in particular, we will propose an alternative decision tree that is more resource efficient than its automatically generated counterparts. For more details refer to the Section 4.2.3.

### 4.2. Task Sequencing Optimization

Once the candidate trees were obtained, our next objective was to determine the optimal strategy for collecting noise pollution data through smartphones. To achieve this purpose, in this section we will analyze the computation time associated with each tree element, as well as its level of accuracy. Secondly, we will analyze the energy consumption required. Finally, we will present our proposal for a balanced tree in terms of computation time and energy savings.

#### 4.2.1. Computation Time

To analyze the computation time associated to each particular task, a specific application was developed to allow evaluating each attribute individually, and following a repeatable and reliable procedure. In general, 100 independent readings were obtained for each attribute to be measured, and we took their average value. A Samsung S7 Edge model running the Android 7.0.2 operating system was used for testing. Below we detail some relevant characteristics of the most critical attributes:

For the *TaskDate, and TaskArea* attributes, the developed application reads the data from an internal database (i.e., SQLite), and then these values were instantiated in a class for later use. We assume that the server application had previously sent these tasks to the smartphone, and so they are available for processing. In particular, the TaskArea attribute was implemented as a class that compares a polygon of n sides with the current position given by the GPS sensor. This class returns true if the smartphone is located inside such polygon.

Regarding the *Speaker, Music,* and *ActiveCall*, attributes, the developed application works by making calls to the corresponding API offered by the operating system. The implementation of the *PhoneStatus* attribute was carried out through a service that reads the proximity, light, and accelerometer sensors. In particular, this service ends when the results are obtained. Notice that we rely on the results of a previous study [18,30] to determine, based on the sensor feedback, whether the smartphone is being held, it is stored in a backpack, or it is in a pocket. So, those previous results allow us to estimate the complexity of such predictions.

The *location* attribute is considered a critical factor because of its high consumption and processing time. To evaluate this attribute, we implemented a service where we read the latitude and longitude of the GPS sensor embedded in the smartphone. Also, we recorded the prediction accuracy to have a greater control of the positions obtained. A time-stamp was recorded when the GPS obtained the first coordinate. In particular, the design of our solutions aims at outdoor locations alone, meaning that we will also use the GPS to discriminate between both cases (indoor vs. outdoor). To assess the ability of the GPS sensor to differentiate between both environments, we first analyzed the accuracy results achieved inside a building (near to a window to get worst-case conditions), as well as outdoors, in an open environment. For each case, 30 records were taken at two different times: mid-morning, and mid-afternoon. Our goal is to check whether the obtained readings through our application show differentiating features for these environments.

**Figure 6:**
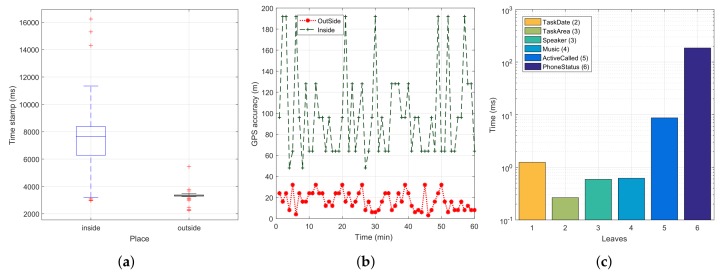
Block size analysis. (**a**) GPS accuracy analysis: indoor vs. outdoor measurements; (**b**) GPS error range; (**c**) Processing time for the different tree elements.

Figure 6a shows that, in outdoor environments, 99% of the location measurements were obtained in less than 4000 ms, with just sporadic values found in the four to six seconds duration range. Besides, to ensure that the smartphone is indeed in an outdoor environment, Figure 6b shows that a GPS accuracy (error) of 40 m or less is typically only obtainable outdoors, while indoors the accuracy (error) is typically much higher, thus allowing to discriminate between both contexts quite easily. Nevertheless, in terms of estimated error, the location API will converge to very low errors in a few seconds only when outdoors, situation where the GPS signal is available to obtain a reliable location fix. Instead, when indoors, the error cannot be reduced due to lack of GPS signal, meaning that it remains high throughout time, as only wireless networks’ data are available to provide a coarse location estimation. Hence, based on these results, we can validate the attribute location in the scope of our tree, and we will set its duration to 4000 ms, as it provides the necessary trade-off between consumption and accuracy. Finally, Figure 6c shows the computation times associated to each key element of the tree (excluding the *Location* parameter). In this Figure, it is noticeable that the *PhoneStatus* attribute is the one consuming more resources in our tree, i.e., 185 ms, followed by the *ActiveCall* attribute, that needs about 9 ms.

Regarding the PhoneStatus attribute, our goal was to determine, with a certain level of accuracy, whether the phone is on the user’s hand (phone either in the normal vertical position, or with left or right turn), or in a desk, but with the front facing upwards. As stated earlier, we have considered these options as the ideal moment to capture environmental noise. The idea of the different states is that there are particular user preferences when used in those positions. The capture of our training dataset was produced as shown in Table 4, and our main goal was to determine the actual phone position: held on the hand, or at a desk facing upwards. We have developed an application to capture the different proposed states in a supervised way. The application reads the sensors: calibrated gyroscope, proximity sensor, linear acceleration, and light sensor. The capture frequency was three samples per second during one minute. After completing our learning set, the data were extracted, and we used Matlab as the tool for the handling and validation of the data.

In particular, we proceeded with the following methodology: (i) we used the K-means algorithm to classify the output from the linear acceleration sensor and the light sensor into three groups. For the linear acceleration, a single value was taken for its three axes; (ii) Once the previous classifications were obtained, a single matrix was made along with the gyroscope values in “x”, “y”, “z”, and the proximity sensor; (iii) Finally, our resulting set was processed using three different classification algorithms, and using the k-fold cross-validation on ten observations. Regarding their accuracy, MatLab shows that the Decision Tree achieves a 99.70% accuracy, while for the linear support vector machines algorithm, the accuracy achieved is slightly reduced (86.20%). The same performance occurs when using the algorithm of discrimination with a 67.90% accuracy. Table 5 shows the confusion matrix when using the Decision Tree. We can recognize that the different states that we want to validate using our tree are clearly differentiated.

#### 4.2.2. Energy Consumption

After determining the computation times associated with each decision attribute, we then proceeded to analyze the energy consumption of the different decisions trees. Our methodology relies on Event-based models [27]. Specifically, a background service was implemented on the smartphone that is periodically reading the different attributes of the proposed tree; for all cases, we set the sampling period to 4 s. Before each test, we check that the smartphone’s battery is at 100%, and that Internet access is disabled. To obtain representative results, the evaluation lasted for 1 h. The smartphone used for testing is the same as above, having a battery capacity of 3600 mAh. In general, three different types of readings were made for comparison, all of them having the smartphone in the suspended mode. The situations under comparison were: (i) without the application installed; (ii) with the developed application running and testing the different attributes (but without activating the location attribute); and (iii) with the application running, but only the attribute that activates the GPS (location) is enabled. The idea of separating the location attribute from the rest is to have a better insight about the values associated with the different elements. Notice that the high amount of time and resources associated with the location attribute would blur the values related to the other attributes (typically lasting less than 200 ms), thus making such measurements less reliable and representative.

Experimental results show that the one-hour consumption estimation without the application running is of 36 mAh. Then, when turning on our developed application and running it in the background, energy consumption increases to 108 mAh. Finally, if the GPS value is obtained through the location attribute, energy consumption further raises to 180 mAh. Based on the measurements made, it was possible to assess the energy consumed (μAh) by each attribute in our tree. The equation used for this purpose is the following:

(1) $$E_{c}=\frac{t_{l}}{\sum t_{l}}.\frac{E_{r}-E_{o}}{N}$$

In this equation, $E_{c}$ represents the energy consumed during 1 s, $t_{l}$ represents the time overhead associated with each tree attribute, and $E_{r}$ and $E_{o}$ represent the reference value for the energy consumed with and without the application, respectively. *N* is the total number of occurrences recorded in an hour. Table 6 summarizes the energy consumption estimation associated with each attribute on the tree. In particular, we can observe that the attributes corresponding to the GPS and PhoneStatus present the highest energy consumption values.

#### 4.2.3. Proposed Tree and Performance Improvement

**Figure 7:**
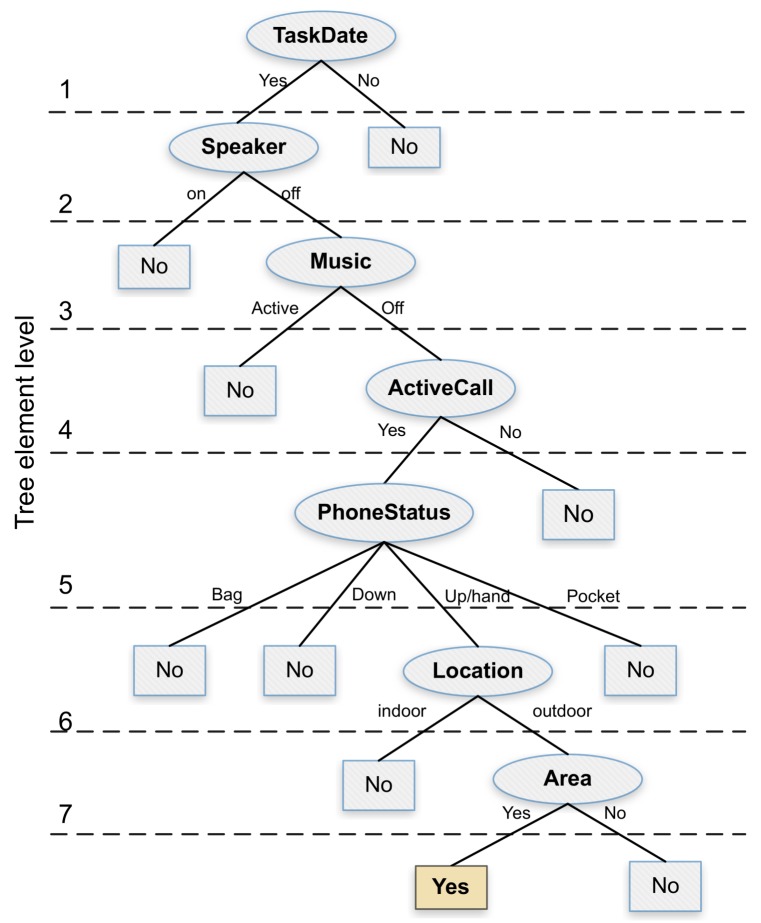
Proposed resource-efficient decision tree.

Once we obtained the computation time overhead and the energy consumption associated with each attribute, our next goal was to propose an alternative decision tree that is more resource efficient. For this purpose, we designed a tree in such a way that its elements are organized and balanced according to the desired objective of reducing time and energy overhead. In particular, for the *TaskDate*, *Speaker*, *Music*, *ActiveCall*, *PhoneStatus*, and *Location* attributes, we followed a sequential order by considering the computation time calculated earlier. Figure 7 shows the proposed decision tree which, similarly to the J48 algorithm, can achieve a decision accuracy of 100%. Notice that, in this alternative tree, the location attribute is located near the tree bottom, thereby optimizing the overall system resources whenever a previous attribute allows discarding the noise sampling procedure by not meeting the required conditions. Besides, we can observe that the area attribute remains just below the location attribute due to its direct dependency, being this an attribute with a lower computation time, but nevertheless highly relevant in terms of the final decision.

**Figure 8:**
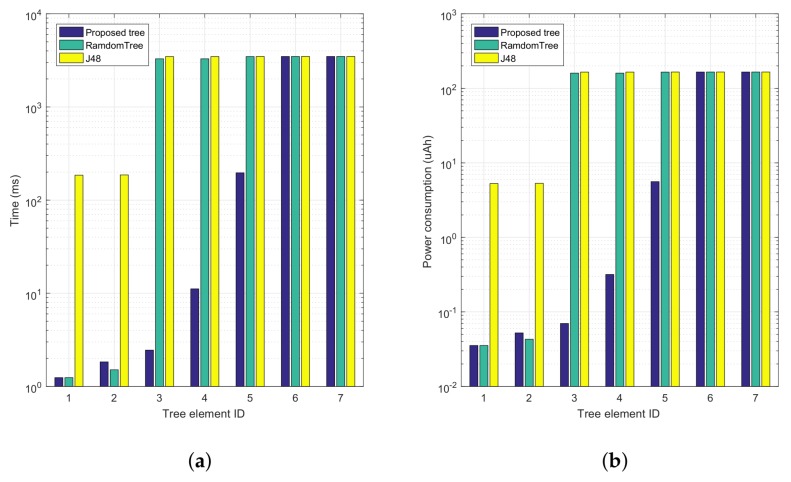
Time and energy consumption corresponding to the different tree levels. (**a**) Time overhead analysis; (**b**) Energy consumption analysis.

To gain further insight into the performance gains achieved, Figure 8 shows a comparison of the accumulative computation time and energy consumption for both our proposed tree and the automatically generated trees. Increasing tree element Ids correspond to progressing along the tree, from top to bottom. In particular, Figure 8a shows that our proposal presents a much lower time overhead compared to the two candidate trees, being that high periods of activity only take place whenever it becomes indispensable (near the bottom of the tree); specifically, the first tree elements introduce a lower time overhead compared to the others. In Figure 8b we find a behaviour that is similar to the previous one, although it now represents the overall energy consumption associated with the proposed and automatically generated trees.

Finally, Table 7 summarizes the performance benefits introduced by our alternative decision tree. In particular, it shows both the accumulated and average values for the time overhead and energy consumption associated with the three decision trees being compared. We find that our tree is 57.81% and 58.70% lower than the RandomTree algorithm regarding computation time and average energy consumption, respectively. For the J48 tree, improvements are further boosted by 60%, while maintaining the same decision accuracy.

Overall, we consider that the process followed in this paper to achieve a tree that is both precise and resource efficient is critical to enable the development of a crowdsensing application aiming at a widespread adoption and usage, a problem to be discussed in the next research steps.

### 4.3. Accurate Ambient Noise Assessment Using Smartphones

Once a decision tree has been proposed that adapts to an acceptable energy consumption, and with a low level of computation, we now focus on the capture of the ambient noise. Based on previous work [17], we analyzed the characteristics that influence the design and implementation of reliable systems for the evaluation of noise pollution levels using smartphones. In particular, we examine the behaviour of three different noise measurement algorithms, and we determine the best approach based on a professional and calibrated class II Sound Level Meter [44]. For each algorithm, we evaluated the effect of different sampling frequencies and block sizes. The algorithms and evaluation methodology are described in more detail in [17]. In the following sections, we will describe the tests performed using different smartphone models, and also tests using similar smartphones.

#### 4.3.1. Analysis Using Different Smartphone models and Calibrations

In this section, we evaluate the accuracy of our measurements when obtaining samples using different smartphone models. All smartphones run the Android 6.1 operating system, and can correctly run the developed application.

**Figure 9:**
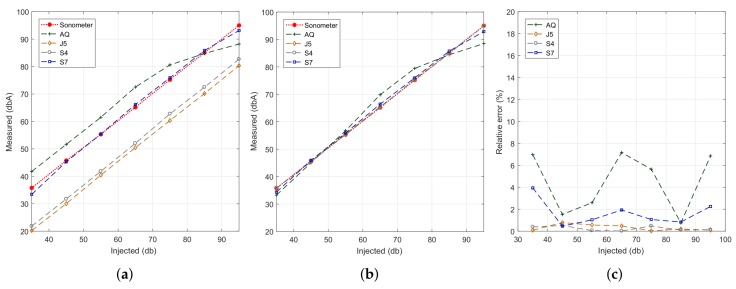
Estimation accuracy for the different smartphone models with and without linear regression. (**a**) default sampling; (**b**) values adjusted using linear regression; (**c**) estimation error.

In our experiments, we injected pink noise in the range from 35 to 95 dB, and each test lasted 30 s. Figure 9a shows the obtained results. In general we find that, except for smartphone model BQ Aquaris (AQ), all other smartphones models (S4, J5 and S7) present a linear behaviour regarding result accuracy; however, we find that only the results for the Samsung S7 device are near the reference values. Such near-optimal accuracy is expected since the experiments performed earlier on relied on this same device. Thus, we find that the results achieved using the proposed algorithm show model-specific variations, which are in general expected due to hardware differences.

To solve the problem detected, our next step was to apply linear regression techniques with respect to the reference dataset; the latter was obtained with the sound level meter at the same time instants. Our goal was to adjust the results achieved with the different smartphones models so that they resemble the reference ones as much as possible. The results of this curve adjustment process are shown in Figure 9b. It quickly becomes evident that the output results for most of the models fully agree with the reference value, with more pronounced differences for the BQ Aquaris smartphones case in the range from 65 to 75 dB (A). Finally, Figure 9c shows that, after the adjustment procedure, the error is less than 2% in Samsung phones, being the BQ Aquaris model the one showing the highest error values. Anyway, the error is always less than 8%, which is a reasonable value.

#### 4.3.2. Analysis for a Same Smartphone Model

**Figure 10:**
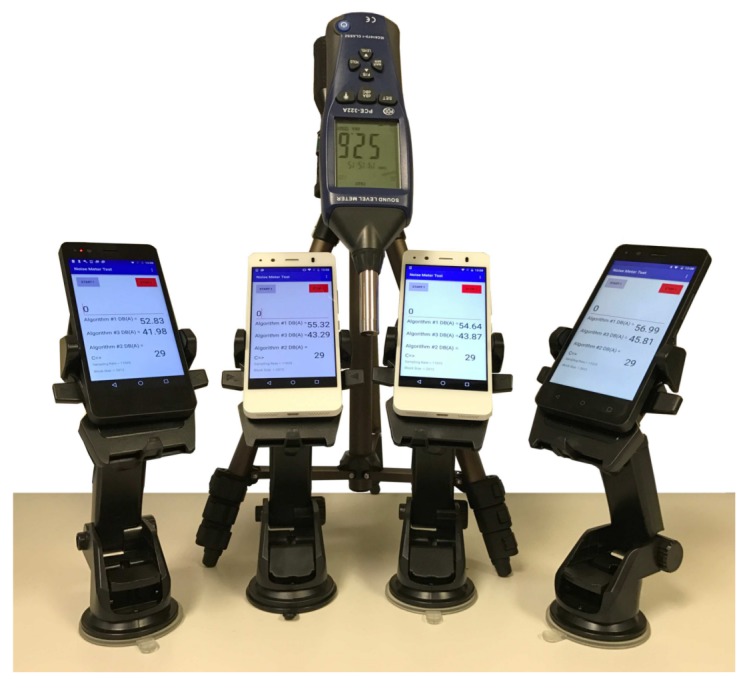
Smartphone same models used for testing.

**Figure 11:**
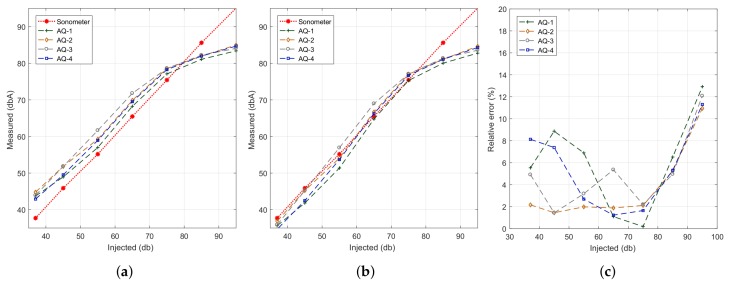
Estimation error analysis when using similar smartphones. (**a**) before regression; (**b**) after regression; (**c**) estimation error.

We now proceed to compare the differences between smartphones of the same model and provider, determining the differences among them. Notice that, in general, differences are expected, especially for low-range market devices, where cheaper hardware is used. Figure 10a shows the smartphones of a same model being evaluated. We picked four BQ Aquaris smartphones for our tests since these are the cheapest ones used. Figure 11a shows the results of our noise-sensing tests before applying any curve adjustment. The experiment was performed under the same conditions detailed earlier. We can see that there are some differences between smartphones, although the shape of the curve is similar in all cases, with a loss of linearity for values above 75 dB. Figure 11b shows the output after performing the linear regression procedure. We can now see how values tend to resemble the reference values better, showing differences for inputs of 85 dB and above due to the non-linearity detected earlier. Figure 11c shows the value of the estimation errors, which are below 8% in most of the cases.

Concerning the variations in the microphones’ sensitivity, it is possible to reduce such variations by modifying the sensitivity levels through programming the gain and the bias voltage [45]. Additionally, we consider that the estimated error in the sensitivity of each individual microphone can be compensated through proper calibration in dedicated environments for noise analysis, such as laboratories endowed with a reverberant acoustic chamber. Another factor that affects the estimated error obtained in measurements is the position and distance of smartphones towards the noise source (injected dB), an issue that must be considered in both indoor and outdoor studies [46,47,48,49].

## 5. Validation of the Proposed Architecture

The current noise-sensing infrastructure, based on professional sonometers, allows measuring noise pollution levels in cities with high accuracy, although with a very low time and spatial resolution due to the limited number of devices available. In contrast, when adopting our proposed crowdsensing approach, we can achieve much higher time and spatial resolution by relying on the microphones of commercial of-the-shelf smartphones.

In a previous work [17] we validated our calibration process in real urban environments. In this section, we validate the effectiveness of our approach in two scenarios: (i) a shopping mall; and (ii) our university campus. The idea of the first scenario is to show that our solution can be used in an environment that is far from the nearest city, and for which there is in general no data regarding noise levels. Concerning the second test scenario, we want to demonstrate that the noise values obtained within our Technical University of Valencia (UPV) campus, in Valencia, Spain, differ from those delivered by the Valencia City council, which takes no actual measurements inside the campus.

**Figure 12:**
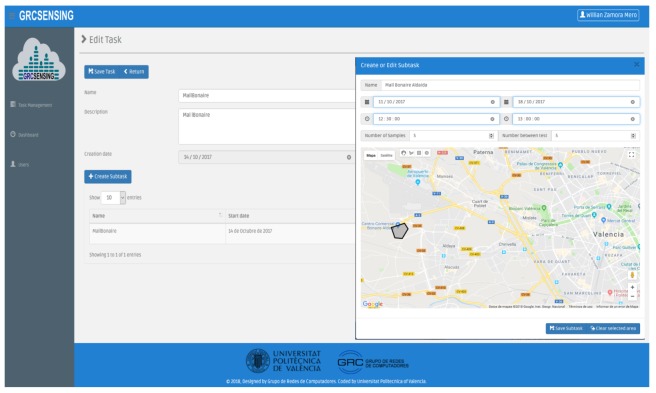
Task specification at the back-end server for noise distribution analysis at the Bonaire shopping mall.

**Figure 13:**
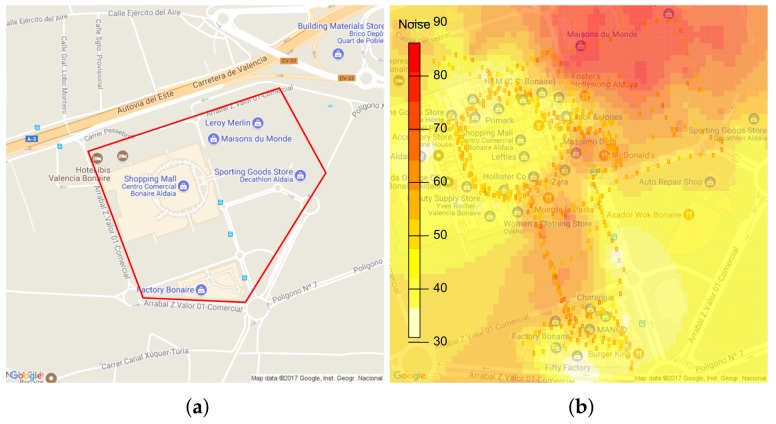
Analysis of the environmental noise in a shopping mall. (**a**) Task specification at the back-end server; (**b**) Noise distribution of the shopping mall (Bonaire).

In the first scenario, we evaluated our proposal in a shopping mall characterized by having free pedestrian circulation and open aisles. To perform this test, we first defined the task on the server, and set it so that the noise capturing takes place during the weekend, specifically on a Saturday from 12:30 to 13:00 PM. The task definition is made through our web platform (http://www.grcsensing.net), and it is shown in Figure 12. Figure 13a shows the coverage area for our first test scenario. Concerning users, this first validation was made with four people who took a random and simultaneous tour on the interior facilities (hallway) and outside of the shopping mall. Each of them used different smartphones with the application installed and activated. Figure 13b shows the result of processing the results at the server to obtain a heat map. It is observed that the places where highest noise values are detected correspond to vehicular parking areas, while in interiors much lower values are measured.

**Figure 14:**
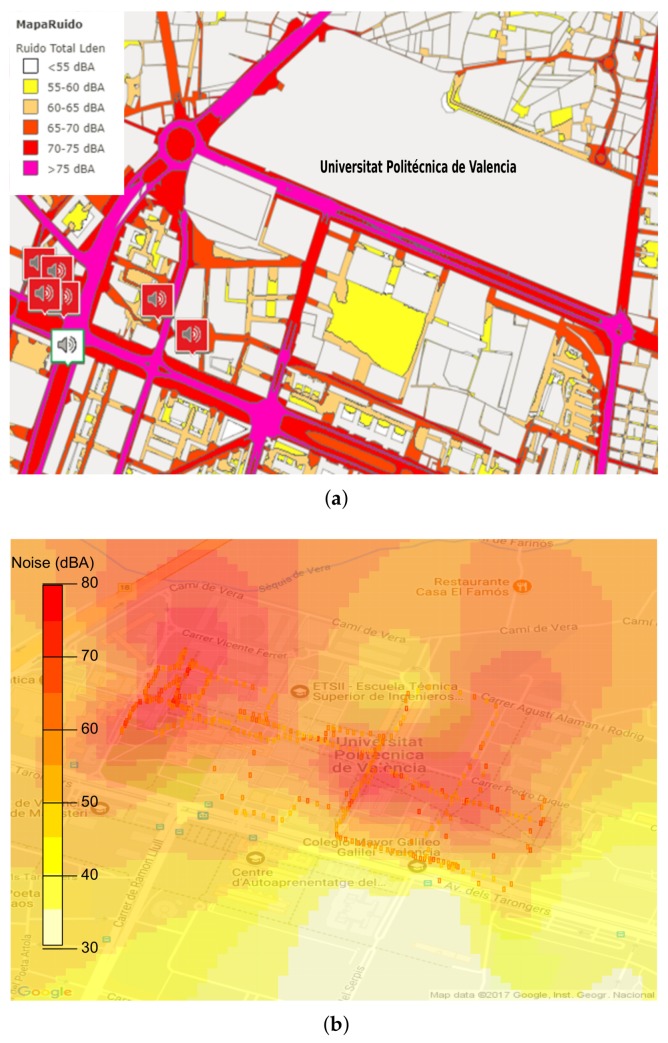
Comparison of the distribution of environmental noise according to official data and using our architecture. (**a**) Analysis of the noise distribution by the city council; (**b**) Analysis of the noise distribution by our proposed approach.

In the second scenario, we evaluated the noise at the UPV campus. The idea of this evaluation is to compare our proposed architecture solution with the noise analysis provided on the website of the City Hall of Valencia [50]. In Figure 14a we show the noise map for the area surrounding the campus as provided by the City Hall. In general, it is noticeable that high noise levels are located in places of high traffic, like main avenues. We can also see that this figure represents with the white colour (<50 dBA) the facilities of our university. Also, the static noise monitoring systems available, which provide constant measurements, are depicted in the figure. As we can see, they are scarce and located at strategic places only.

In view of the above, we proceeded to generate a sensing task on the server covering an hour during which many students gather to take a break or coffee (between 17:15 to 17:45 p.m.). Specifically, we define a target area sized 800 × 600 square meters. In particular, this test was performed with two people simultaneously walking, one at each end, allowing to cover the interior parts of the campus. The smartphones used were Samsung S7 devices. Figure 14b shows the results obtained. In general, when compared with Figure 14a, we find a very representative difference concerning the noise values found during that hour of the day, especially regarding data within the campus. Also, the heat map shown in Figure 14b indicates a high-level noise zone in areas near the coffee shop and restaurants around the campus. Regarding the actual amount of data read on smartphones, the Samsung G930F had a 99.94% effectiveness regarding noise sampling opportunities, while the Samsung G935F device had an effectiveness of 94.5%. In the latter, the value varied because in a small part of his test the user walked through areas covered with a ceiling, thereby failing GPS accuracy tests. Overall, the total number of samples was 382.

Finally, these results show that our crowdsensing solution for environmental noise analysis can benefit different public or private institutions by providing a real-time data with high spatial and temporal granularity. Based on the information obtained, authorities can detect locations where noise limits are not within the bounds regulated by local laws, and better plan the development in areas of high environmental risk, including the distribution of bus lines, the location of leisure areas, among others. Also, our solution provides an easy interface for the administrator to define tasks, and visualize the outcome of such tasks in the form of heat maps, allowing to see how noise evolves throughout time, and thus assess if any corrective measures taken were effective, and to which extent.

## 6. Conclusions and Future Work

Currently, crowdsensing solutions have become an enabling technology for Smart Cities by empowering users to participate in the monitoring process of their environment through their mobile devices. In this scope, studies of noise pollution over densely populated areas is not an exception, with different works pointing in this direction.

In this paper, we proposed a complete architecture for environmental noise monitoring that combines smartphones and cloud services to measure noise pollution levels with high spatial granularity. In detail, we use smartphones as mobile sensors to provide noise pollution measurements, and rely on Firebase as a gateway technology, allowing the interaction between the sending of sensing tasks at back-end servers, and the noise capture (by smartphones) at client devices. Once the task is delivered, the smartphone decides the optimum time for capturing data, and it provides real-time feedback on the given noise quality conditions; finally, the back-end server provides services for storage, processing, and data visualization.

Once the architecture was defined, we analyzed different issues related to the sampling process: (i) An attribute set to determine the ideal context conditions for noise sampling, based on which we obtained candidate decision trees; (ii) The computation time required for each node of the candidate trees and the energy consumed was analyzed; (iii) A proposal was made for a balanced tree in terms of computing and energy overhead associated with each attribute; and (iv) The impact of having different types of smartphones on noise measurements was analyzed. Experimental results show that a theoretical classification does not necessarily provide an optimal decision tree regarding computation overhead and energy consumption. In particular, our proposal obtained relative savings of nearly 60% regarding both energy consumption and computing overhead. Concerning the quality of the measurements gathered, we find some differences between smartphones, even those from a same model, although these differences are in general negligible.

Finally, we validated our architecture evidencing that, compared to static solutions for environmental noise monitoring, it can provide a much higher time and spatial granularity. As future work, we plan to add new functionalities that include minimizing the number of users required to cover specific areas by exploiting Firebase features for real-time communications.

## Figures and Tables

**Table 1:** Characteristics of crowdsensing-based noise assessment platforms.

Publication	Smartphones			Data Transmission Networks	Cloud Server Task Specification		
	Noise Calibration Procedure	Data Collection	Inteligent Context Awareness		Selection Area/Date	Real Time Data Task	Share Maps
NoiseTube [9]	Linear interpolation	Participative interaction	No	offline	Manual	No	Yes
NoiseSPY [10]	Correlation calibrated	Participative	No	online (cache store)	Manual	No	No
NoiseMap [31]	Few details	Participative	No detail	offline	No detail	No	Yes
NoiseHound [32]	Few details	Participative	Strategy area for spatio-temporal	Offline ad hoc nework	No	No	Yes
Usense [20]	No-detail	Automatic by Server	Yes Rule-based attributes	No detail	Automatic Area and between date	Yes	No detail
WideNoise [33]	No detail	Participative	No detail	Offline	Manual area	No	Yes
NoizCrowd [12]	No detail	Participative	No	Offline	No detail task area	No	Yes
SoundOfTheCity [13]	No detail	Participative	Yes	Offline (SOAP)	No	No	Yes
Sense2health [34]	Few details	Participative sensing	No detail	Yes (publish/subscribe RabbitMQ)	No	No	Yes
Ear-Phone [11,18]	Regression lineal	Participative	Yes	No	No	No	Unclear
OnoM@p [35]	Cross calibration	Participatory	No detail	No detail	No details	No	Yes
Soundscape@p [21]	Few details	Participatory	Yes	Online	No details	No	Yes
GRC-Sensing	Regression lineal and others	Automatic Only Install	Yes	Online	Yes	Yes	Yes

**Table 2:** Size of the message with different selection area and data captured noise.

Data JSON	Description Type	Message Size (Bytes)
DataNoise	Normal data	236
DataTask	With a polygon of 5 points	363
	With a polygon of 10 points	404
	With a polygon of 20 points	411
	With a circle (unencoded)	461
	With a rectangle (unencoded )	509

**Table 3:** Attribute candidates according to their category.

Categories	Attribute
Task	TaskDate, TaskArea
Sound	Speaker, Music, ActiveCall, MicroPhone, ActiveApplication
Status	PhoneStatus, Blocked, Camera, Keyboard, Location

**Table 4:** Details of the training dataset.

Label	Status	Orientation	Movement	Response	Total Dataset
Hand (1)	Left, Vertical, Right	North, South, West, East	Static/Walking	X	4320
Pocket and bag (2)	Up/Down; Vertical/Horizontal	North	Static/Walking		1440
Desk up (3)	Up	North, South, West, East	Static	X	720
Desk down (4)	Down	North, South, West, East	Static		720

**Table 5:** Confusion matrix associated to phone status recognition.

Label	Recognized Value			
	(1)	(2)	(3)	(4)
Hand (1)	4310	9	1	-
Pocket and bag (2)	5	1434	-	1
Desk up (3)	2	-	718	719
Desk down (4)	-	2	-	718

**Table 6:** Energy consumption estimation for each tree leaf.

Tree Element	Random Tree (μ Ah)	J48 (μ Ah)
1	0.0354	5.2761
2	0.0076	0.0354
3	160.00	160.00
4	0.0168	0.0076
5	5.2761	0.2479
6	0.2479	0.0176
7	0.0176	0.0168

**Table 7:** Estimation of computational requirements and energy consumption for the different decision trees.

Total	Algorithm	CPU Time (ms)		Energy (μAh)	
# Elements		∑	$\bar{\sum }$	∑	$\bar{\sum }$
7	Proposed tree	3483.89	897.72	165.60	42.16
7	RandomTree	3483.89	2127.84	165.60	102.09
7	J48	3483.89	2244.44	165.60	105.41

## Abbreviations

The following abbreviations are used in this paper:

GPS	Global Positioning System
CPU	Central Processor Unit
MNSC	Mobile Noise-Sensing Client
CDC	Cloud Data Colletion
CUI	Client User Interface
CSTM	Client Sensor Task Manager
CDM	Client Data Manager
CCM	Client Communications Manager (CCM)
STM	Server Task Manager
SDM	Server Data Manager
SCM	Server Communications Manager
SED	Software Event Detector
API	Application Programming Interface
JSON	JavaScript Object Notation
SQL	Structured Query Language
RESTful	Representational State Transfer
SC	Sensor Controller
UPV	Technical University of Valencia
